# Benzyl­tributyl­ammonium 6-hydroxy­naphthalene-2-sulfonate

**DOI:** 10.1107/S1600536809001329

**Published:** 2009-01-17

**Authors:** Kazuya Uta, Yohei Sato, Jin Mizuguchi

**Affiliations:** a Department of Applied Physics, Graduate School of Engineering, Yokohama National University, 79-5 Tokiwadai, Hodogaya-ku, 240-8501 Yokohama, Japan

## Abstract

The title compound, C_19_H_34_N^+^·C_10_H_7_O_4_S^−^, is a charge-control agent for toners used in electrophotography. Inter­moleclar O—H⋯O hydrogen bonding between the OH group of one anion and the sulfonate O atom of a neighboring anion leads to the formation of one-dimensional chains along the *b* axis. In addition, C—H⋯O hydrogen bonds are observed. One of the *n*-butyl chains of the cation is disordered over two sites in a 0.88:0.12 ratio.

## Related literature

For general background to charge-control agents for toners, see: Nash *et al.* (2001 ▶). For a related structure, see: Mizuguchi *et al.* (2007 ▶).
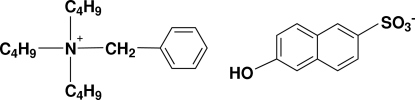


## Experimental

### 

#### Crystal data

C_19_H_34_N^+^·C_10_H_7_O_4_S^−^
*M* *_r_* = 499.70
Monoclinic, 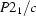

*a* = 16.9616 (4) Å
*b* = 10.4422 (2) Å
*c* = 17.6700 (4) Å
β = 116.2570 (11)°
*V* = 2806.73 (11) Å^3^
*Z* = 4
Cu *K*α radiation
μ = 1.28 mm^−1^
*T* = 296.1 K
0.50 × 0.25 × 0.04 mm

#### Data collection

Rigaku R-AXIS RAPID diffractometer
Absorption correction: multi-scan (*ABSCOR*; Higashi, 1995 ▶) *T*_min_ = 0.720, *T*_max_ = 0.954
24483 measured reflections
5103 independent reflections
2818 reflections with *F* ^2^ > 2σ(*F* ^2^)
*R*_int_ = 0.032

#### Refinement

*R*[*F* ^2^ > 2σ(*F* ^2^)] = 0.055
*wR*(*F* ^2^) = 0.191
*S* = 1.10
5103 reflections
328 parameters
H-atom parameters constrained
Δρ_max_ = 0.27 e Å^−3^
Δρ_min_ = −0.40 e Å^−3^

### 

Data collection: *PROCESS-AUTO* (Rigaku, 1998 ▶); cell refinement: *PROCESS-AUTO*; data reduction: *CrystalStructure* (Rigaku/MSC, 2006 ▶); program(s) used to solve structure: *SIR2004* (Burla *et al.*, 2003 ▶); program(s) used to refine structure: *SHELXL97* (Sheldrick, 2008 ▶); molecular graphics: *ORTEPIII* (Burnett & Johnson, 1996 ▶); software used to prepare material for publication: *CrystalStructure*.

## Supplementary Material

Crystal structure: contains datablocks global, I. DOI: 10.1107/S1600536809001329/ci2755sup1.cif

Structure factors: contains datablocks I. DOI: 10.1107/S1600536809001329/ci2755Isup2.hkl

Additional supplementary materials:  crystallographic information; 3D view; checkCIF report

## Figures and Tables

**Table 1:** Hydrogen-bond geometry (Å, °)

*D*—H⋯*A*	*D*—H	H⋯*A*	*D*⋯*A*	*D*—H⋯*A*
O4—H4*O*⋯O2^i^	0.82	1.88	2.696 (3)	177
C12—H12*B*⋯O2^ii^	0.96	2.52	3.470 (4)	173
C16—H16*B*⋯O3^i^	0.98	2.34	3.251 (4)	155

Symmetry codes: (i) 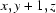; (ii) 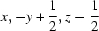.

**Fig. 1.**
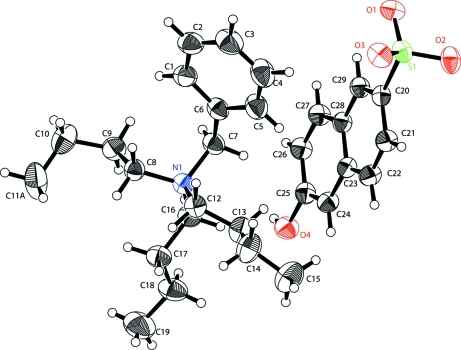
The asymmetric unit of the title compound. Displacement ellipsoids are drawn at the 30% probability level. Only the major disorder component is shown.

**Fig. 2.**
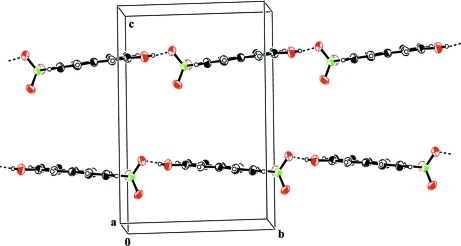
The formation of a hydrogen-bonded (dashed lines) chain. Only anions are shown for clarity.

## supplementary crystallographic information

### Comment

Quaternary ammonium salts (for example, benzyltributylammonium 4-hydroxynaphthalene-1-sulfonate: P-51 from Orient Chemical Industries) are well known charge-control agents (CCAs) for toners used in electrophotography. CCAs are usually added to toners to create a desired charge level and polarity (Nash *et al.*, 2001). The above compounds are characterized by high melting point above 433 K. The present high thermal stability is required for the toner manufacturing process which includes kneading of various toner components such as polymer, colorant, wax and CCA at 403–453 K. However, ordinary quaternary ammonium salts used in electrochemistry as supporting electrolytes exhibit much lower melting points below 373 K. Previously, we have investigated why P-51 alone possesses such a high melting point from the standpoint of the crystal structure. Then, we found chains of O—H···O intermolecular hydrogen bonds between the OH group of one anion and the sulfonate O atom of the neighboring one (Mizuguchi *et al.*, 2007). The formation of the hydrogen bond is found to be responsible for the high thermal stability of P-51. A s an extension of this study, the present paper deals with the structure of the title compound, which is one of the P-51 derivatives.

Fig. 1 shows the *ORTEPIII* plot (Burnett & Johnson, 19966) of the title molecule. The ions have no crystallographically imposed symmetry. Fig. 2 shows a hydrogen-bonded anionic chain along the *b* axis between the OH group of one anion and the sulfonate O atom of the neighboring one. In addition, C—H···O hydrogen bonds are observed in the crystal structure (Table 1). The hydrogen-bonding network is found to greatly contribute to the high melting point of the title compound (433 K), just as in the case of P-51 (462 K).

### Experimental

The title compound was obtained from Orient Chemical Industries Ltd., and was recrystallized from a methanol solution. After 48 h, a number of colourless crystals were obtained in the form of platelets.

### Refinement

Atom C11 was found to be disordered over two sites. The site occupancies for C11A/C11B were initially refined and later fixed at 0.88/0.12. These atoms were anisotropically refined. All H atoms were placed in geometrically idealized positions and constrained to ride on their parent atoms, with C—H = 0.93 Å (aromatic), 0.96 Å (methyl), or 0.97 Å (methylene), and O—H = 0.82 Å; *U*_iso_(H) = 1.2–1.5*U*_eq_(parent atom).

### Crystal data

**Table:**

C_19_H_34_N^+^·C_10_H_7_O_4_S^−^	*F*(000) = 1080.00
*M**_r_* = 499.70	*D*_x_ = 1.183 Mg m^−^^3^
Monoclinic, *P*2_1_/*c*	Cu *K*α radiation, λ = 1.54187 Å
Hall symbol: -P 2ybc	Cell parameters from 15945 reflections
*a* = 16.9616 (4) Å	θ = 3.0–68.2°
*b* = 10.4422 (2) Å	µ = 1.28 mm^−^^1^
*c* = 17.6700 (4) Å	*T* = 296 K
β = 116.2570 (11)°	Plate, colourless
*V* = 2806.73 (11) Å^3^	0.50 × 0.25 × 0.04 mm
*Z* = 4	

### Data collection

**Table:**

Rigaku R-AXIS RAPID diffractometer	2818 reflections with *F*^2^ > 2σ(*F*^2^)
Detector resolution: 10.00 pixels mm^-1^	*R*_int_ = 0.032
ω scans	θ_max_ = 68.2°
Absorption correction: multi-scan (*ABSCOR*; Higashi, 1995)	*h* = −20→20
*T*_min_ = 0.720, *T*_max_ = 0.954	*k* = −12→12
24483 measured reflections	*l* = −21→21
5103 independent reflections	

### Refinement

**Table:**

Refinement on *F*^2^	H-atom parameters constrained
*R*[*F*^2^ > 2σ(*F*^2^)] = 0.055	*w* = 1/[σ^2^(*F*_o_^2^) + (0.07*P*)^2^ + 1.689*P*] where *P* = (*F*_o_^2^ + 2*F*_c_^2^)/3
*wR*(*F*^2^) = 0.191	(Δ/σ)_max_ = 0.001
*S* = 1.10	Δρ_max_ = 0.27 e Å^−^^3^
5103 reflections	Δρ_min_ = −0.40 e Å^−^^3^
328 parameters	

### Special details

**Table:**

Geometry. All e.s.d.'s (except the e.s.d. in the dihedral angle between two l.s. planes) are estimated using the full covariance matrix. The cell e.s.d.'s are taken into account individually in the estimation of e.s.d.'s in distances, angles and torsion angles; correlations between e.s.d.'s in cell parameters are only used when they are defined by crystal symmetry. An approximate (isotropic) treatment of cell e.s.d.'s is used for estimating e.s.d.'s involving l.s. planes.
Refinement. Refinement of *F*^2^ against ALL reflections. The weighted *R*-factor *wR* and goodness of fit *S* are based on *F*^2^, conventional *R*-factors *R* are based on *F*, with *F* set to zero for negative *F*^2^. The threshold expression of *F*^2^ > σ(*F*^2^) is used only for calculating *R*-factors(gt) *etc*. and is not relevant to the choice of reflections for refinement. *R*-factors based on *F*^2^ are statistically about twice as large as those based on *F*, and *R*- factors based on ALL data will be even larger.

### Fractional atomic coordinates and isotropic or equivalent isotropic displacement parameters (Å^2^)

**Table:**

	*x*	*y*	*z*	*U*_iso_*/*U*_eq_	Occ. (<1)
S1	0.72863 (6)	−0.06500 (8)	0.72643 (5)	0.0673 (2)	
O1	0.64931 (16)	−0.0460 (2)	0.73670 (15)	0.0775 (6)	
O2	0.79375 (17)	−0.1418 (2)	0.79490 (15)	0.0840 (7)	
O3	0.71380 (17)	−0.1121 (2)	0.64457 (14)	0.0805 (7)	
O4	0.89003 (16)	0.6746 (2)	0.76763 (17)	0.0837 (7)	
N1	0.71413 (18)	0.5740 (2)	0.48646 (16)	0.0636 (6)	
C1	0.5395 (2)	0.3823 (4)	0.4426 (2)	0.0829 (10)	
C2	0.4932 (2)	0.2707 (5)	0.4115 (2)	0.0948 (12)	
C3	0.5324 (3)	0.1552 (5)	0.4400 (3)	0.1058 (14)	
C4	0.6187 (3)	0.1490 (4)	0.4993 (3)	0.1091 (15)	
C5	0.6659 (2)	0.2610 (4)	0.5289 (2)	0.0922 (12)	
C6	0.6279 (2)	0.3792 (3)	0.5014 (2)	0.0729 (9)	
C7	0.6794 (2)	0.4989 (3)	0.5397 (2)	0.0729 (9)	
C8	0.6398 (2)	0.6301 (3)	0.4083 (2)	0.0691 (8)	
C9	0.5816 (2)	0.7256 (3)	0.4246 (2)	0.0849 (10)	
C10	0.5138 (3)	0.7839 (4)	0.3429 (3)	0.1152 (14)	
C11A	0.5529 (5)	0.8743 (7)	0.3027 (4)	0.145 (3)	0.88
C11B	0.499 (2)	0.774 (4)	0.2541 (10)	0.120 (8)	0.12
C12	0.7657 (2)	0.4880 (3)	0.4554 (2)	0.0701 (9)	
C13	0.8520 (2)	0.4368 (4)	0.5213 (2)	0.0877 (11)	
C14	0.8937 (3)	0.3471 (4)	0.4819 (3)	0.1095 (14)	
C15	0.9840 (3)	0.3085 (6)	0.5412 (4)	0.148 (2)	
C16	0.7723 (2)	0.6785 (3)	0.5429 (2)	0.0736 (9)	
C17	0.8068 (2)	0.7742 (3)	0.5005 (2)	0.0821 (10)	
C18	0.8740 (3)	0.8608 (4)	0.5651 (2)	0.1039 (14)	
C19	0.8973 (4)	0.9751 (5)	0.5287 (3)	0.142 (2)	
C20	0.7782 (2)	0.0872 (2)	0.73747 (19)	0.0591 (7)	
C21	0.8585 (2)	0.0989 (3)	0.7323 (2)	0.0686 (8)	
C22	0.8973 (2)	0.2154 (3)	0.7391 (2)	0.0711 (9)	
C23	0.8581 (2)	0.3286 (3)	0.75144 (19)	0.0607 (7)	
C24	0.8940 (2)	0.4517 (3)	0.7557 (2)	0.0662 (8)	
C25	0.8535 (2)	0.5573 (3)	0.7660 (2)	0.0657 (8)	
C26	0.7749 (2)	0.5462 (3)	0.7738 (2)	0.0679 (8)	
C27	0.7390 (2)	0.4290 (3)	0.7700 (2)	0.0694 (8)	
C28	0.7778 (2)	0.3164 (2)	0.75807 (19)	0.0600 (7)	
C29	0.7396 (2)	0.1939 (3)	0.7505 (2)	0.0637 (8)	
H1	0.5117	0.4606	0.4249	0.107*	
H2	0.4349	0.2744	0.3713	0.121*	
H3	0.5001	0.0806	0.4187	0.134*	
H4	0.6455	0.0700	0.5191	0.138*	
H4O	0.8599	0.7308	0.7740	0.134*	
H5	0.7246	0.2558	0.5688	0.117*	
H7A	0.7295	0.4774	0.5924	0.094*	
H7B	0.6427	0.5573	0.5532	0.094*	
H8A	0.6026	0.5591	0.3754	0.092*	
H8B	0.6643	0.6697	0.3744	0.092*	
H9A	0.6195	0.7924	0.4617	0.110*	
H9B	0.5529	0.6833	0.4544	0.110*	
H10A	0.4837	0.7156	0.3035	0.138*	0.88
H10B	0.4704	0.8296	0.3544	0.138*	0.88
H10C	0.4579	0.7594	0.3413	0.138*	0.12
H10D	0.5190	0.8753	0.3539	0.138*	0.12
H11A	0.5899	0.8275	0.2843	0.217*	0.88
H11B	0.5871	0.9381	0.3430	0.217*	0.88
H11C	0.5066	0.9151	0.2551	0.217*	0.88
H11D	0.4736	0.6916	0.2320	0.180*	0.12
H11E	0.5535	0.7830	0.2511	0.180*	0.12
H11F	0.4590	0.8400	0.2214	0.180*	0.12
H12A	0.7296	0.4148	0.4277	0.090*	
H12B	0.7775	0.5346	0.4148	0.090*	
H13A	0.8416	0.3936	0.5644	0.112*	
H13B	0.8904	0.5097	0.5475	0.112*	
H14A	0.8585	0.2700	0.4617	0.144*	
H14B	0.8968	0.3876	0.4337	0.144*	
H15A	1.0082	0.2512	0.5147	0.233*	
H15B	0.9832	0.2656	0.5898	0.233*	
H15C	1.0206	0.3832	0.5609	0.233*	
H16A	0.8213	0.6398	0.5893	0.096*	
H16B	0.7382	0.7251	0.5667	0.096*	
H17A	0.8322	0.7307	0.4688	0.104*	
H17B	0.7582	0.8275	0.4611	0.104*	
H18A	0.8514	0.8896	0.6034	0.128*	
H18B	0.9261	0.8096	0.5965	0.128*	
H19A	0.9420	1.0211	0.5738	0.226*	
H19B	0.8478	1.0235	0.4979	0.226*	
H19C	0.9225	0.9420	0.4920	0.226*	
H21	0.8853	0.0268	0.7241	0.087*	
H22	0.9508	0.2212	0.7367	0.091*	
H24	0.9464	0.4604	0.7511	0.085*	
H26	0.7479	0.6192	0.7816	0.089*	
H27	0.6870	0.4229	0.7760	0.089*	
H29	0.6873	0.1859	0.7549	0.083*	

### Atomic displacement parameters (Å^2^)

**Table:**

	*U*^11^	*U*^22^	*U*^33^	*U*^12^	*U*^13^	*U*^23^
S1	0.0846 (6)	0.0493 (4)	0.0677 (5)	−0.0029 (4)	0.0336 (4)	−0.0039 (3)
O1	0.0811 (15)	0.0723 (15)	0.0930 (17)	−0.0088 (12)	0.0512 (13)	−0.0066 (12)
O2	0.1071 (18)	0.0523 (13)	0.0804 (16)	0.0101 (13)	0.0304 (14)	0.0063 (11)
O3	0.1080 (18)	0.0711 (15)	0.0679 (14)	−0.0156 (13)	0.0438 (13)	−0.0229 (12)
O4	0.0858 (16)	0.0560 (14)	0.1050 (19)	−0.0103 (12)	0.0383 (14)	−0.0062 (13)
N1	0.0717 (16)	0.0652 (17)	0.0576 (15)	−0.0036 (13)	0.0320 (13)	−0.0055 (13)
C1	0.081 (2)	0.090 (2)	0.080 (2)	−0.012 (2)	0.038 (2)	−0.003 (2)
C2	0.091 (2)	0.104 (3)	0.087 (2)	−0.029 (2)	0.036 (2)	−0.012 (2)
C3	0.127 (4)	0.093 (3)	0.095 (3)	−0.036 (3)	0.047 (3)	−0.016 (2)
C4	0.135 (4)	0.074 (2)	0.102 (3)	−0.013 (2)	0.037 (3)	0.005 (2)
C5	0.104 (3)	0.079 (2)	0.080 (2)	−0.011 (2)	0.029 (2)	0.003 (2)
C6	0.089 (2)	0.071 (2)	0.0592 (19)	−0.013 (2)	0.0332 (18)	−0.0043 (17)
C7	0.084 (2)	0.078 (2)	0.062 (2)	−0.0089 (19)	0.0364 (18)	−0.0029 (17)
C8	0.073 (2)	0.073 (2)	0.0627 (19)	0.0024 (17)	0.0304 (17)	0.0008 (16)
C9	0.086 (2)	0.085 (2)	0.094 (2)	0.009 (2)	0.048 (2)	0.002 (2)
C10	0.097 (3)	0.115 (3)	0.115 (3)	0.029 (2)	0.030 (2)	−0.003 (2)
C11A	0.181 (6)	0.158 (6)	0.108 (4)	0.075 (4)	0.077 (4)	0.051 (4)
C11B	0.106 (16)	0.120 (18)	0.102 (5)	0.004 (16)	0.017 (11)	0.009 (13)
C12	0.079 (2)	0.070 (2)	0.066 (2)	0.0034 (18)	0.0360 (18)	−0.0059 (17)
C13	0.088 (2)	0.094 (2)	0.076 (2)	0.014 (2)	0.032 (2)	−0.001 (2)
C14	0.103 (3)	0.107 (3)	0.105 (3)	0.029 (2)	0.034 (2)	−0.003 (2)
C15	0.106 (3)	0.157 (5)	0.168 (5)	0.037 (3)	0.050 (3)	0.019 (4)
C16	0.081 (2)	0.071 (2)	0.070 (2)	−0.0148 (18)	0.0341 (18)	−0.0136 (18)
C17	0.088 (2)	0.079 (2)	0.084 (2)	−0.016 (2)	0.041 (2)	−0.015 (2)
C18	0.116 (3)	0.102 (3)	0.102 (3)	−0.043 (2)	0.055 (2)	−0.025 (2)
C19	0.157 (4)	0.117 (4)	0.144 (4)	−0.057 (3)	0.060 (3)	−0.012 (3)
C20	0.0646 (18)	0.0539 (18)	0.0538 (16)	0.0011 (14)	0.0217 (14)	−0.0009 (14)
C21	0.071 (2)	0.057 (2)	0.080 (2)	0.0049 (16)	0.0356 (18)	−0.0071 (16)
C22	0.0642 (19)	0.062 (2)	0.084 (2)	0.0008 (16)	0.0307 (18)	−0.0062 (17)
C23	0.0586 (17)	0.0566 (19)	0.0607 (18)	0.0021 (15)	0.0208 (14)	−0.0031 (14)
C24	0.0625 (19)	0.057 (2)	0.073 (2)	−0.0037 (15)	0.0250 (16)	−0.0088 (16)
C25	0.072 (2)	0.0512 (19)	0.0646 (19)	−0.0051 (16)	0.0217 (16)	−0.0048 (15)
C26	0.078 (2)	0.0503 (19)	0.078 (2)	0.0024 (16)	0.0371 (18)	−0.0069 (16)
C27	0.074 (2)	0.0540 (19)	0.085 (2)	0.0023 (16)	0.0389 (18)	−0.0032 (17)
C28	0.0641 (18)	0.0514 (18)	0.0622 (18)	0.0035 (14)	0.0259 (15)	−0.0006 (14)
C29	0.0685 (19)	0.0508 (18)	0.073 (2)	0.0033 (15)	0.0320 (16)	0.0022 (15)

### Geometric parameters (Å, °)

**Table:**

S1—O1	1.449 (3)	C5—H5	0.933
S1—O2	1.465 (2)	C7—H7A	0.969
S1—O3	1.441 (2)	C7—H7B	0.972
S1—C20	1.768 (3)	C8—H8A	0.981
O4—C25	1.367 (4)	C8—H8B	0.961
N1—C7	1.530 (5)	C9—H9A	0.978
N1—C8	1.517 (3)	C9—H9B	0.967
N1—C12	1.515 (5)	C10—H10A	0.970
N1—C16	1.513 (4)	C10—H10B	0.970
C1—C2	1.376 (6)	C10—H10C	0.970
C1—C6	1.398 (4)	C10—H10D	0.970
C2—C3	1.361 (7)	C11A—H11A	0.960
C3—C4	1.375 (6)	C11A—H11B	0.960
C4—C5	1.383 (6)	C11A—H11C	0.960
C5—C6	1.377 (5)	C11B—H11D	0.960
C6—C7	1.503 (5)	C11B—H11E	0.960
C8—C9	1.518 (6)	C11B—H11F	0.960
C9—C10	1.519 (5)	C12—H12A	0.966
C10—C11A	1.500 (10)	C12—H12B	0.959
C10—C11B	1.48 (2)	C13—H13A	0.966
C12—C13	1.509 (4)	C13—H13B	0.975
C13—C14	1.515 (7)	C14—H14A	0.971
C14—C15	1.478 (6)	C14—H14B	0.974
C16—C17	1.513 (6)	C15—H15A	0.957
C17—C18	1.507 (5)	C15—H15B	0.973
C18—C19	1.490 (8)	C15—H15C	0.962
C20—C21	1.410 (5)	C16—H16A	0.960
C20—C29	1.362 (4)	C16—H16B	0.982
C21—C22	1.362 (5)	C17—H17A	0.959
C22—C23	1.419 (5)	C17—H17B	0.983
C23—C24	1.410 (4)	C18—H18A	0.961
C23—C28	1.423 (5)	C18—H18B	0.969
C24—C25	1.355 (5)	C19—H19A	0.952
C25—C26	1.406 (6)	C19—H19B	0.923
C26—C27	1.354 (4)	C19—H19C	0.983
C27—C28	1.408 (5)	C21—H21	0.924
C28—C29	1.413 (4)	C22—H22	0.930
O4—H4O	0.817	C24—H24	0.932
C1—H1	0.926	C26—H26	0.930
C2—H2	0.930	C27—H27	0.936
C3—H3	0.931	C29—H29	0.928
C4—H4	0.931		
			
O2···O4^i^	2.696 (3)	C11B···H2^iv^	2.896
O4···O2^ii^	2.696 (3)	C25···H7A	2.970
C2···C11B^iii^	2.99 (3)	H2···O1^v^	2.993
C11B···C2^iv^	2.99 (3)	H2···O3^v^	2.947
S1···H4O^i^	2.925	H2···C11B^iii^	2.896
O1···H2^v^	2.993	H3···O1^v^	2.817
O1···H3^v^	2.817	H4···O3	2.757
O1···H8A^vi^	2.893	H4O···S1^ii^	2.925
O1···H8B^vi^	2.665	H4O···O2^ii^	1.880
O1···H10B^vii^	2.989	H7A···C25	2.970
O1···H12B^vi^	2.930	H8A···O1^ix^	2.893
O2···H4O^i^	1.880	H8B···O1^ix^	2.665
O2···H12B^vi^	2.516	H9B···C1^vii^	2.961
O2···H17A^vi^	2.992	H9B···C2^vii^	2.846
O2···H26^i^	2.593	H10B···O1^vii^	2.989
O3···H2^v^	2.947	H11D···C2^iv^	2.949
O3···H4	2.757	H11E···C2^iv^	2.624
O3···H16B^i^	2.336	H11F···C1^iv^	2.942
O3···H18A^i^	2.736	H11F···C2^iv^	2.895
O4···H15B^viii^	2.661	H12B···O1^ix^	2.930
O4···H16A	2.859	H12B···O2^ix^	2.516
O4···H22^viii^	2.777	H15B···O4^x^	2.661
C1···H9B^vii^	2.961	H16A···O4	2.859
C1···H11F^iii^	2.942	H16B···O3^ii^	2.336
C2···H9B^vii^	2.846	H17A···O2^ix^	2.992
C2···H11D^iii^	2.949	H18A···O3^ii^	2.736
C2···H11E^iii^	2.624	H22···O4^x^	2.777
C2···H11F^iii^	2.895	H26···O2^ii^	2.593
			
O1—S1—O2	111.98 (17)	C9—C10—H10B	108.9
O1—S1—O3	114.40 (15)	C9—C10—H10C	104.0
O1—S1—C20	106.62 (16)	C9—C10—H10D	103.9
O2—S1—O3	112.04 (15)	C11A—C10—H10A	108.9
O2—S1—C20	104.67 (13)	C11A—C10—H10B	108.9
O3—S1—C20	106.32 (17)	C11B—C10—H10C	103.9
C7—N1—C8	111.5 (2)	C11B—C10—H10D	103.9
C7—N1—C12	111.1 (2)	H10A—C10—H10B	107.7
C7—N1—C16	106.3 (2)	H10C—C10—H10D	105.4
C8—N1—C12	106.2 (2)	C10—C11A—H11A	109.5
C8—N1—C16	111.0 (2)	C10—C11A—H11B	109.5
C12—N1—C16	110.7 (2)	C10—C11A—H11C	109.5
C2—C1—C6	120.8 (3)	H11A—C11A—H11B	109.5
C1—C2—C3	120.3 (3)	H11A—C11A—H11C	109.5
C2—C3—C4	120.3 (4)	H11B—C11A—H11C	109.5
C3—C4—C5	119.4 (4)	C10—C11B—H11D	109.5
C4—C5—C6	121.5 (3)	C10—C11B—H11E	109.5
C1—C6—C5	117.6 (3)	C10—C11B—H11F	109.5
C1—C6—C7	122.3 (3)	H11D—C11B—H11E	109.5
C5—C6—C7	119.9 (2)	H11D—C11B—H11F	109.5
N1—C7—C6	117.0 (3)	H11E—C11B—H11F	109.5
N1—C8—C9	115.4 (3)	N1—C12—H12A	108.4
C8—C9—C10	111.4 (3)	N1—C12—H12B	108.4
C9—C10—C11A	113.3 (4)	C13—C12—H12A	107.0
C9—C10—C11B	133.1 (18)	C13—C12—H12B	107.8
N1—C12—C13	116.7 (3)	H12A—C12—H12B	108.5
C12—C13—C14	110.8 (3)	C12—C13—H13A	108.7
C13—C14—C15	113.0 (4)	C12—C13—H13B	107.8
N1—C16—C17	115.6 (3)	C14—C13—H13A	111.0
C16—C17—C18	110.5 (3)	C14—C13—H13B	110.5
C17—C18—C19	114.2 (3)	H13A—C13—H13B	107.9
S1—C20—C21	119.8 (2)	C13—C14—H14A	110.2
S1—C20—C29	120.9 (2)	C13—C14—H14B	110.5
C21—C20—C29	119.4 (3)	C15—C14—H14A	108.0
C20—C21—C22	120.9 (3)	C15—C14—H14B	107.4
C21—C22—C23	121.1 (3)	H14A—C14—H14B	107.4
C22—C23—C24	123.1 (3)	C14—C15—H15A	111.0
C22—C23—C28	117.9 (3)	C14—C15—H15B	109.7
C24—C23—C28	119.0 (3)	C14—C15—H15C	109.6
C23—C24—C25	121.0 (3)	H15A—C15—H15B	108.7
O4—C25—C24	118.7 (3)	H15A—C15—H15C	109.6
O4—C25—C26	120.9 (3)	H15B—C15—H15C	108.2
C24—C25—C26	120.4 (3)	N1—C16—H16A	109.0
C25—C26—C27	119.6 (3)	N1—C16—H16B	107.7
C26—C27—C28	122.2 (3)	C17—C16—H16A	108.8
C23—C28—C27	117.7 (3)	C17—C16—H16B	108.4
C23—C28—C29	119.2 (3)	H16A—C16—H16B	107.2
C27—C28—C29	123.1 (3)	C16—C17—H17A	110.4
C20—C29—C28	121.5 (3)	C16—C17—H17B	109.7
C25—O4—H4O	109.9	C18—C17—H17A	110.0
C2—C1—H1	119.9	C18—C17—H17B	108.2
C6—C1—H1	119.3	H17A—C17—H17B	107.9
C1—C2—H2	119.7	C17—C18—H18A	108.5
C3—C2—H2	120.0	C17—C18—H18B	107.1
C2—C3—H3	119.3	C19—C18—H18A	108.6
C4—C3—H3	120.4	C19—C18—H18B	109.9
C3—C4—H4	120.4	H18A—C18—H18B	108.4
C5—C4—H4	120.2	C18—C19—H19A	107.9
C4—C5—H5	118.8	C18—C19—H19B	110.3
C6—C5—H5	119.7	C18—C19—H19C	106.2
N1—C7—H7A	107.2	H19A—C19—H19B	113.4
N1—C7—H7B	107.0	H19A—C19—H19C	108.2
C6—C7—H7A	109.2	H19B—C19—H19C	110.6
C6—C7—H7B	108.9	C20—C21—H21	119.8
H7A—C7—H7B	107.2	C22—C21—H21	119.3
N1—C8—H8A	107.8	C21—C22—H22	119.8
N1—C8—H8B	108.6	C23—C22—H22	119.1
C9—C8—H8A	107.9	C23—C24—H24	119.5
C9—C8—H8B	109.4	C25—C24—H24	119.5
H8A—C8—H8B	107.4	C25—C26—H26	119.8
C8—C9—H9A	107.8	C27—C26—H26	120.6
C8—C9—H9B	109.2	C26—C27—H27	118.8
C10—C9—H9A	110.3	C28—C27—H27	119.0
C10—C9—H9B	110.4	C20—C29—H29	119.3
H9A—C9—H9B	107.5	C28—C29—H29	119.2
C9—C10—H10A	108.9		
			
O1—S1—C20—C21	178.7 (2)	C8—C9—C10—C11A	−70.7 (5)
O1—S1—C20—C29	−1.7 (2)	C8—C9—C10—C11B	−6(2)
O2—S1—C20—C21	59.9 (3)	N1—C12—C13—C14	177.0 (3)
O2—S1—C20—C29	−120.5 (2)	C12—C13—C14—C15	171.9 (4)
O3—S1—C20—C21	−58.9 (2)	N1—C16—C17—C18	−171.6 (3)
O3—S1—C20—C29	120.8 (2)	C16—C17—C18—C19	−167.4 (4)
C7—N1—C8—C9	61.7 (3)	S1—C20—C21—C22	178.9 (2)
C8—N1—C7—C6	65.9 (3)	S1—C20—C29—C28	−178.9 (2)
C7—N1—C12—C13	−68.0 (3)	C21—C20—C29—C28	0.7 (4)
C12—N1—C7—C6	−52.5 (3)	C29—C20—C21—C22	−0.8 (4)
C7—N1—C16—C17	−173.9 (3)	C20—C21—C22—C23	−0.2 (4)
C16—N1—C7—C6	−173.0 (2)	C21—C22—C23—C24	−177.5 (3)
C8—N1—C12—C13	170.5 (3)	C21—C22—C23—C28	1.3 (4)
C12—N1—C8—C9	−177.1 (3)	C22—C23—C24—C25	178.8 (3)
C8—N1—C16—C17	−52.4 (4)	C22—C23—C28—C27	−179.8 (2)
C16—N1—C8—C9	−56.6 (4)	C22—C23—C28—C29	−1.3 (4)
C12—N1—C16—C17	65.3 (3)	C24—C23—C28—C27	−1.1 (4)
C16—N1—C12—C13	49.8 (4)	C24—C23—C28—C29	177.5 (2)
C2—C1—C6—C5	−2.0 (7)	C28—C23—C24—C25	0.1 (3)
C2—C1—C6—C7	−177.1 (4)	C23—C24—C25—O4	−178.4 (2)
C6—C1—C2—C3	2.3 (8)	C23—C24—C25—C26	0.8 (4)
C1—C2—C3—C4	−0.7 (9)	O4—C25—C26—C27	178.5 (3)
C2—C3—C4—C5	−1.0 (9)	C24—C25—C26—C27	−0.8 (4)
C3—C4—C5—C6	1.2 (9)	C25—C26—C27—C28	−0.2 (4)
C4—C5—C6—C1	0.3 (7)	C26—C27—C28—C23	1.1 (4)
C4—C5—C6—C7	175.5 (5)	C26—C27—C28—C29	−177.4 (3)
C1—C6—C7—N1	−79.8 (5)	C23—C28—C29—C20	0.3 (4)
C5—C6—C7—N1	105.2 (4)	C27—C28—C29—C20	178.8 (3)
N1—C8—C9—C10	175.5 (3)		

Symmetry codes: (i) *x*, *y*−1, *z*; (ii) *x*, *y*+1, *z*; (iii) −*x*+1, *y*−1/2, −*z*+1/2; (iv) −*x*+1, *y*+1/2, −*z*+1/2; (v) −*x*+1, −*y*, −*z*+1; (vi) *x*, −*y*+1/2, *z*+1/2; (vii) −*x*+1, −*y*+1, −*z*+1; (viii) −*x*+2, *y*+1/2, −*z*+3/2; (ix) *x*, −*y*+1/2, *z*−1/2; (x) −*x*+2, *y*−1/2, −*z*+3/2.

### Hydrogen-bond geometry (Å, °)

**Table:**

*D*—H···*A*	*D*—H	H···*A*	*D*···*A*	*D*—H···*A*
O4—H4O···O2^ii^	0.82	1.88	2.696 (3)	177
C12—H12B···O2^ix^	0.96	2.52	3.470 (4)	173
C16—H16B···O3^ii^	0.98	2.34	3.251 (4)	155

Symmetry codes: (ii) *x*, *y*+1, *z*; (ix) *x*, −*y*+1/2, *z*−1/2.
